# Quantifying the evidence and burden of smoking behaviour on tuberculosis incidence among adult population: a systematic review and meta-analysis

**DOI:** 10.7189/jogh.16.04079

**Published:** 2026-03-06

**Authors:** Wenmei Zhao, Wai Yan Min Htike, Yiu-Wing Kam

**Affiliations:** Division of Natural and Applied Science, Duke Kunshan University, Suzhou, China

## Abstract

**Background:**

Tuberculosis (TB) remains a major public health challenge in China and worldwide, with smoking being a key modifiable risk factor. Given China’s large population and rising smoking rates, this paper aims to examine the link between smoking and TB incidence.

**Methods:**

We systematically searched six databases from inception for studies reporting smoking exposure, TB outcomes, and smoker-non-smoker comparisons. Two reviewers independently screened records, extracted data, and assessed bias. We analysed smoking-TB associations using random-effects meta-analysis of odds ratios (ORs) and hazard ratios (HRs).

**Results:**

We included 17 studies reporting ORs and 7 studies reporting HRs in the quantitative synthesis. The pooled OR for TB incidence among smokers compared with non-smokers was 1.77 (95% confidence interval (CI) = 1.29–2.43), indicating a statistically significant increase in risk of TB. For studies reporting hazard ratios, the pooled estimate was 2.39 (95% CI = 1.28–4.45), showing a significant association between smoking and increased TB incidence.

**Conclusions:**

Both active and passive smoking significantly elevate the risk of TB and worsen its outcomes in China. Our result indicate that COVID-19 pandemic may have indirectly exacerbated smoking-related risks through disruptions to TB services, heightened psychosocial stress, and shifts in smoking behaviours, with potential implications for TB risk and outcomes. Thus, integrating smoking cessation strategies into TB programmes, focusing on heavy smokers in especially high-prevalence areas, and raising public awareness could enhance efforts to prevent and control TB worldwide.

**Registration:**

PROSPERO: CRD420251070123.

Tuberculosis (TB) continues to represent a critical global public health challenge, with smoking identified as one of the key modifiable risk factors. In 2023, there were a total of 1.25 million deaths attributed to TB, alongside an estimated 10.8 million new cases, comprising 6.0 million men, 3.6 million women, and 1.3 million children [1]. To achieve the global targets established at the 2023 United Nations High-Level Meeting on TB, approximately USD 22 billion in annual funding is required for prevention, diagnosis, treatment, and care [1]. Importantly, these financial requirements extend not only to the global scale but are particularly crucial for low- and middle-income countries. Between 2000 and 2017, total TB-related health expenditure across 135 low- and middle-income countries increased by an average of 3.9% annually, rising from USD 5.7 billion in 2000 to USD 10.9 billion in 2017 [2]. Despite gradual reductions in TB-related mortality since 2000, TB deaths have consistently exceeded those caused by HIV, a severe disease known to compromise essential components of the immune system and some other infectious diseases [3]. In 2023, an estimated 1.25 million people died from TB infection compared with approximately 630 000 deaths attributed to HIV [1]. However, the COVID-19 pandemic disrupted this trend. In 2021, the global number of people who developed TB increased to an estimated 10.6 million, marking a 4.5% rise compared to the previous year [4]. This reversal underscores the fragility of progress in TB control and the urgent need for sustained international commitment and resources.

In China, TB continues to pose a significant public health concern, despite substantial reductions in incidence and mortality over the past three decades [5]. According to the Global Burden of Disease Study (2021), TB accounts for 0.25% of China’s total disability-adjusted life years (DALYs), despite an annual decline of 2.86% [6]. Meanwhile, as the world’s largest producer and consumer of tobacco, with around 50% of adult men being current smokers and slightly smaller female populations, China also bears a disproportionately high prevalence of smoking and its associated burden of morbidity and mortality [7]. The combined challenges of TB-related mortality and widespread tobacco use underscore the urgency of addressing TB as a critical global health priority.

The urgency of addressing TB stems from its impact not only at the regional level but also globally. The World Health Organization’s (WHO) post-2015 End TB Strategy [8], endorsed by the World Health Assembly in 2014 [9], was designed to align with the Sustainable Development Goals and aims to eliminate the global TB epidemic. The strategy established ambitious targets that by 2030, TB-related deaths should be reduced by 90% compared with 2015 levels, while TB incidence should be reduced by 80% [9]. Despite notable progress in reducing mortality rates and saving millions of lives, significant challenges remain. These include economic constraints, inequities in health system distribution, and the persistent burden of HIV-associated TB. In this context, China presents a particularly pressing case. As a country with both high TB incidence and one of the world’s highest rates of tobacco consumption [10], it is critical to consider potential associations between widespread smoking behaviours and the burden of TB. Given China’s large population and rising smoking prevalence [11], addressing TB in China, therefore, has important implications not only for national public health but also for global TB control efforts.

To address this gap, we conducted a systematic review and meta-analysis to examine the impact of smoking behaviour on TB incidence, focusing specifically on the relationship between smoking and TB risk in China.

## METHODS

### Search strategy and selection criteria

We conducted a systematic review following PRISMA 2020 guidelines [12] and registered the protocol on PROSPERO (CRD420251070123). We performed a comprehensive search across 6 electronic databases (PubMed, Embase, MEDLINE, Web of Science, PsycINFO, and Cochrane Library) from inception to June 2025. The search combined three core concept groups related to TB, smoking, and population terms (Table S1 in the **Online Supplementary Document**). Two reviewers (WZ and WYMH) independently screened all retrieved records at two stages (title/abstract and full-text). Any disagreements were resolved through discussion, and when consensus could not be reached, a third reviewer (YWK) was consulted to adjudicate [13]. We also reviewed the reference lists of included studies and relevant reviews to identify additional eligible publications. When multiple reports drew on overlapping study populations, we included the most comprehensive, recent version, prioritising those with the largest sample size, longest follow-up, or most complete reporting of outcomes.

We included six different types of studies: prospective and observational cohort studies, randomised controlled trials/experimental studies, cross-sectional studies, retrospective cohort studies, and case-control studies. Furthermore, we included studies if they met the following criteria [14]: conducted in Chinese populations (either within China or among Chinese populations abroad); included adult participants (≥18 years), regardless of TB status; reported original research data; examined the association between smoking behaviour and active TB incidence; reported TB incidence outcomes, with or without stratification by demographic or clinical characteristics.

We excluded studies involving inappropriate designs (*e.g.* reviews, commentaries, case reports), that were conducted in non-Chinese populations, did not examine smoking as an exposure, did not clearly define TB outcomes, lacked relevant comparator groups, had inaccessible full texts, or had insufficient outcome data.

### Data analysis

We extracted data using a standardised form in Microsoft Excel, version 16.106.02 (Microsoft, Redmond, Washington, USA) including study characteristics (author, year, location, sample size, design), participant demographics, exposure definitions (smoking categories), outcome measures (TB incidence or diagnosis), and effect estimates (odds ratios (ORs), relative risks (RRs), or hazard ratios (HRs), with 95% confidence intervals (CIs). When multiple effect estimates were reported, we prioritised the model that was most fully adjusted.

Given variability in smoking exposure definitions across studies, we harmonised exposure categories by extracting effect estimates comparing any smoking exposure *vs.* no smoking wherever possible. When studies reported multiple smoking categories, such as current, former, or intensity-based measures, we selected the estimate most comparable to an ever-smoker *vs.* never-smoker contrast.

The primary outcome was the association between smoking and active TB incidence. Preplanned subgroup analyses considered stratification by sex, age, smoking intensity, and study design. Risk of bias in observational studies was assessed using the Newcastle-Ottawa Scale [15]. We resolved disagreements by consensus.

TB incidence definitions varied across studies and included microbiologically confirmed, clinically diagnosed, and radiographically diagnosed TB. We did not exclude studies based on a diagnostic approach, as all included studies defined active TB according to contemporaneous national or WHO-aligned criteria. To address potential heterogeneity arising from diagnostic variation, we applied a random-effects model and explored heterogeneity through sensitivity analyses. No formal reclassification of diagnostic categories was undertaken due to limited reporting detail and the frequent use of composite diagnostic definitions.

To harmonise smoking categories across studies, we prioritised current *vs.* non-current smoking as the primary exposure. We combined former smokers with never smokers unless separately reported. For studies reporting multiple intensity levels, we used the highest exposure category or the most comparable category across studies. Furthermore, we classified TB outcomes according to study definitions; for pooling, we prioritised confirmed TB cases, while we grouped broader TB definitions separately to ensure comparability across studies.

As this review included heterogeneous observational designs, including case-control and retrospective cohort studies, we anticipated methodological differences and potential sources of bias. To address this, we conducted stratified analyses by study design, random-effects models, and risk-of-bias assessments to account for differences in study populations and measurement methods. Where appropriate, we pooled effect estimates using a random-effects model (DerSimonian-Laird method) to account for heterogeneity [16]. We quantified heterogeneity using the *I*^2^ statistic and further explored sources of variability through sensitivity analyses (*e.g.* exclusion of studies at high risk of bias). We assessed publication bias using funnel plots and Egger’s test, where ≥10 studies were available. We performed all analyses in *R*, version 4.3.1. (R Core Team, Vienna, Austria). We generated a geographical distribution of the 29 selected studies in China, with details on the number of studies for each province, using Datawrapper.

## RESULTS

**Figure 1:**
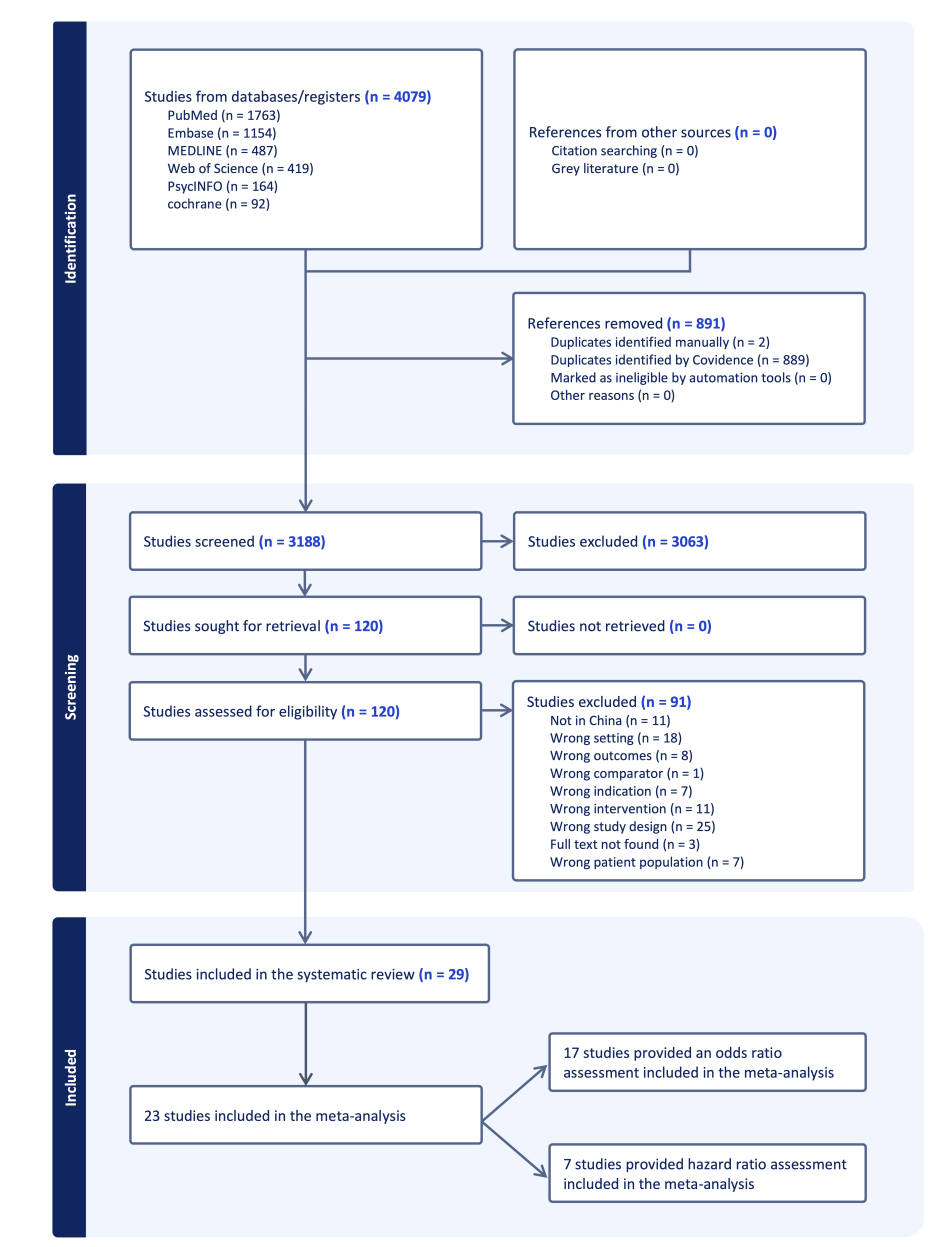
Prisma flow diagram of literature search and study selection

**Figure 2:**
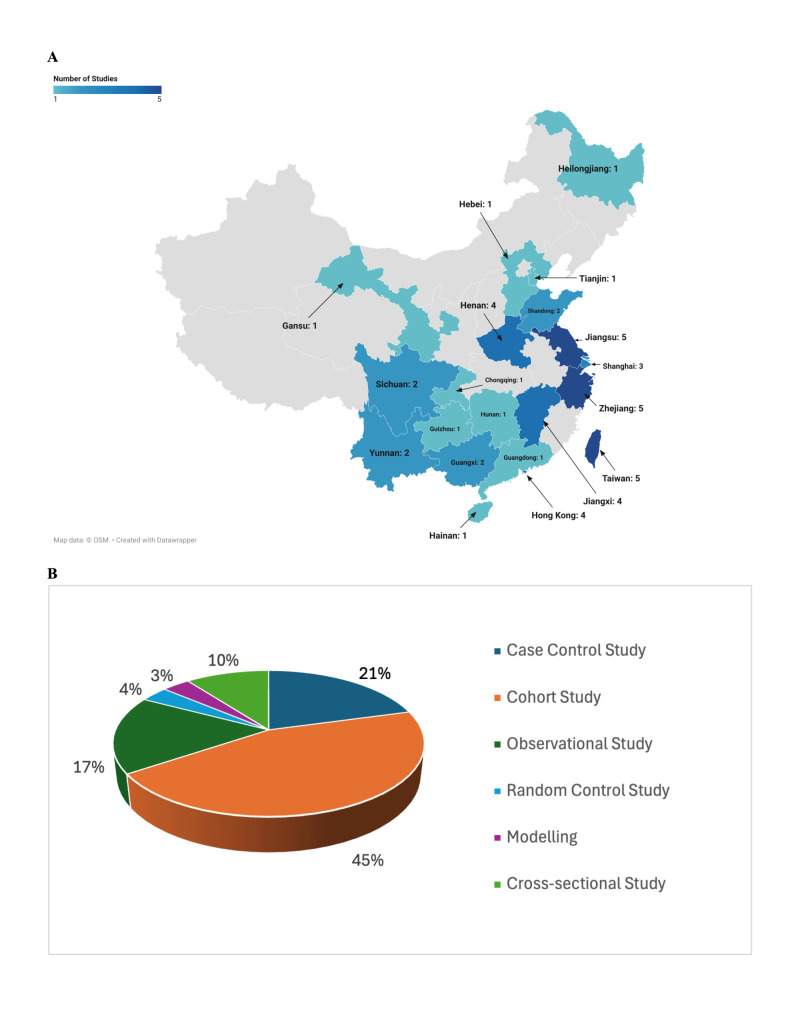
Geographic distribution and study design characteristics of the 29 included studies in China. **Panel A**. Details on the number of studies per province. A thematic map depicting the geographic distribution of the included studies by province was generated using Datawrapper. The map visualises the number of studies conducted in each specific province of China. Grey areas indicate provinces with no studies. The colour scale ranges from 1 to 5, corresponding to the number of studies conducted in each province, where 1 represents the lowest number and 5 the highest. The map of China for this study includes Hong Kong, Macao, and Taiwan. **Panel B**. Distribution of study designs among the included studies. Proportional representation of the study designs included in the systematic review and meta-analysis of smoking behaviour and tuberculosis (TB) incidence in China (n = 29). The ‛Modeling’ category refers to studies that applied a modelled framework based on existing data.

The combined search yielded 4079 publications (1763 from PubMed, 1154 from Embase, 487 from MEDLINE, 419 from Web of Science, 164 from PsycINFO, and 92 from Cochrane Library) between 1997 and 2024. After removing 891 duplicates in Covidence (**Figure 1**), we screened 3188 records by title/abstract and excluded 3063. Of 120 full-text articles assessed, 91 were excluded due to location, setting, or other reasons, resulting in a total of 29 studies included for data extraction. Over half were published after 2010. All 29 studies were conducted in China (**Figure 2**, Panel A, **Table 1**), predominantly in its eastern part (Jiangsu, n = 5; Zhejiang, n = 5; Shanghai, n = 3), followed by Taiwan (n = 5) and Hong Kong (n = 4). We observed moderate representation in midwestern provinces, including Sichuan (n = 2), Yunnan (n = 2), and Guangxi (n = 2). The distribution indicates higher research activity and greater awareness of smoking behaviour in the eastern region of China compared to the western region. For this paper, the eastern region comprises Heilongjiang, Tianjin, Hebei, Shandong, Henan, Jiangsu, Shanghai, Zhejiang, Jiangxi, Hunan, Hainan, Guangdong, Hong Kong, and Taiwan. In contrast, the western region includes Gansu, Sichuan, Yunnan, Guizhou, Guangxi, and Chongqing.

**Table 1:** Characteristics of included studies on the association between smoking and tuberculosis in China

Study, year, reference	Study design	Location	Population	Smoking exposure (definition, type)	ORs/HRs (95% CI)	Follow-up year	Adjustment factor	Study result
KO *et al.*, 1997, [17]	Case-control study	Taiwan	n = 125; adults (0.0% male); age range: (18, +)	≥1 cigarette/ a year for ≥1 year	4.70 (1.50–14.71)	NA	Age, sex, smoking, alcohol, occupational exposure, chronic bronchitis, diet, fuel/oil use, passive smoking	The study confirmed the well-established association between smoking and TB
Wei *et al.*, 2024, [18]	Retrospective study	Sichuan	n = 288; adults (54.5% male); age range: (14, +)	≥20 packs lifetime or ≥1/day for ≥1 year	0.28 (0.12–0.66)	12	Age, sex, ethnicity, TB history, comorbidities, laboratory/imaging, antituberculosis regimen	Current smoking was identified as an independent risk factor for adverse outcomes
Lin *et al.*, 2021, [19]	Prospective cohort study	Jiangxi	n = 634; adults (69.9% male); age range: (14, +)	An ever-smoker who was still smoking during the past year	10.42 (2.97–36.53) *	7	Age, sex, TB type, lung cavities, smoking status/intensity, treatment, HIV, SES	Smoking status was associated with TB recurrence and is modulated by TB type
Leung *et al.*, 2004, [20]	Prospective cohort study	Hong Kong SAR	n = 42 655; Adults (34.8% male); Age range: (65, +)	Regular smoker or ≥6 months lifetime	2.87 (2.00–4.11) *	1	Residential area	Both current smokers and ex-smokers were found to have a higher risk of TB than never-smokers
Feng *et al.*, 2023, [21]	Retrospective case-control study	Southwest China	n = 917; adults (with mean age of 45.2 (cases) and 58.1 (controls) (64.3% male); age range: (18, +)	Smoked ≥6 months ago	3.84 (1.45–10.18)	1	Age, sex, BMI, CCI, hypoproteinaemia, smoking severity	Positive tubercle bacilli in sputum and severe PTB were much higher in smoking patients
Tsai *et al.*, 2016, [22]	Cross-sectional retrospective study	Chiayi, Taiwan	n = 123; adults (78.0% male); age range: (20, +)	Smoker at the time of TB diagnosis	NA	1	Age, sex, diabetes, drug resistance, radiography, smoking	Smokers were 2–3 times more likely to develop TB than non-smokers
Bai *et al.*, 2016, [23]	Retrospective cohort study	Taiwan (Eastern, Northern, Southern)	n = 972; adults (34.7% male); age range: (18, +)	Current smoker (with cessation info)	NA	5	Age, sex, comorbidities, BMI, alcohol, smoking, and drug resistance	Smoking harmed TB outcomes
Lin *et al.*, 2024, [24]	Prospective cohort study	Jiangxi	n = 800; adults (71.5% male); age range: (18, +)	>100 cigarettes lifetime; ≥1 in past 30 day = current	5.40 (3.02–9.66) *	7	Education, BMI, adherence, smoking/drinking type, healthcare access	The study confirmed the finding that smoking is associated with extensive lung disease and cavitary lesions, but these were only observed in non-diabetic and not in diabetic TB patients
Song *et al.*, 2022, [25]	Retrospective cohort study	Shandong	n = 7992; all age groups (63.6% male); age range: (0, +)	≥6 months (current or quit <6 months); ex = quit ≥6 months	0.98 (0.80–1.21)	16	Smoking, passive smoking, diet, fuel/oil, and industrial exposure	Although we did not find an independent impact of alcohol drinking or tobacco smoking on TB drug-resistance, respectively, these two habits had a combined effect on TB drug-resistance
Ma *et al.*, 2019, [26]	Retrospective cohort study	Tianjin, Chongqing, Shanghai, Guangdong, Guangxi, Hebei, Henan, and Yunnan	n = 1256; age ≥15 years (72.9% male); age range: (15, +)	Ever *vs*. never (≥5 cigarettes/month); includes intensity	NA	3	Chronic bronchitis, TB history, SES, alcohol, fuel use	The results demonstrated that both smokers and drinkers among TB patients had a higher proportion
Zhang *et al.*, 2017, [27]	Prospective cross-sectional study	Southwest, south, east, central, northwest, northeast parts of China	n = 21 008; age ≥5 years (46.3% male); age range: (5, +)	≥1 cigarette/day for >6 months	1.34 (1.21–1.49)	0.33	Age, sex, education, smoking, alcohol, TB severity	Populations at high risk of infection, such as elderly smokers, should be controlled prior to TB infection under the premise of community-level intervention
Wang *et al.*, 2022, [28]	Prospective observational study	Zhejiang	n = 1856; rural adults aged ≥65 years (49.5% male); age range: (65, +)	Never ≤5/month; current = ≥5/month	1.43 (1.04-1.97)	1	Age, comorbidities, TB symptoms, diabetes, education, employment	Smoking (current) increases LTBI risk by 43% (aOR = 1.43). Higher LTBI in men (40.6% *vs.* 29.4% in women). Higher LTBI in frequenters of closed spaces (*e.g.* chess rooms: 39.5%)
Xin *et al.*, 2019, [29]	Prospective randomised controlled trial with cross-sectional analysis	Zhongmu County, Zhengzhou	n = 20 486; rural residents aged 50–70 years old (44.6% male); age range: (50, 70)	Smoker at screening (current)	1.40 (1.26–1.55)	0.25	QFT+, BCG scar, smoking, age	A total of 20 486 individuals aged 50 to 70 y were included in the analysis and 4259 (20.79%) of them were diagnosed with MTB infection. Males, heavy smokers and participants with a history of HBV/HCV infection, previous TB or silicosis were found to be more vulnerable to MTB infection
Wei *et al.*, 2015, [18]	Prospective quasi-experimental study	Yunnan	n = 471; smokers attending routine primary care facilities in para-urban communities in Yunnan, China, with additional high-risk factors for TB (93.2% male); age range: (18, +)	Current, former, never (self-report)	6.003 (1.06–34.08)	1	Age, VDD, BMI, cirrhosis, smoking	Higher TB risk among smokers with diabetes (OR = 6.003; 95% CI = 1.057–34.075). Previous studies showed smokers have 1.6–2.8 × higher TB risk than non-smokers
Zhang *et al.*, 2023, [7]	Cross-sectional study	Shandong, Jiangsu, Zhejiang, Hunan, Henan, Sichuan, Hainan, Guangxi, Gansu, Heilongjiang	n = 3494; adults (93.8%); mean age of non-COPD is SD = 57.6–9.6; mean age of COPD is SD = 63.7–10.3	>100 cigarettes lifetime	1.40 (1.26–1.56)	1	Age, rurality, COPD, hepatitis, smoking	The results provided further evidence to support that smoking might increase host susceptibility to TB infection. Populations at high risk of infection, such as elderly smokers, should be controlled prior to TB infection under the premise of community-level intervention
Xiao *et al.*, 2022, [30]	Prospective cohort study	Shanghai	n = 767; school students (freshmen/sophomores) (55.8% male); age range: (15, +)	History of smoking (no definition)	3.92 (1.51–10.17)	2	Smoking, age, hypoalbuminemia, and liver disease	School close contacts should be targeted for LTBI screening and preventive treatment; smoking control is crucial in TB prevention
Hsu *et al.*, 2024, [31]	Prospective age/sex-matched case-control study	Taiwan	n = 310; adults (>20 years) from clinical settings in Northern Taiwan (71.0% male); age range: (21, 89)	Smoked ≥3 months at diagnosis	4.52 (1.85–11.02)	1	Smoking, age, gender, BMI, exercise	Vitamin D deficiency was an independent risk factor for TB; smoking and low BMI were also significant
Ye *et al.*, 2023, [32]	Retrospective case-control study	Wenzhou, Zhejiang Province, China	n = 1062; hospitalised pulmonary TB patients (65.5% male); age range (14, +)	Current smoker only	2.77 (1.77–4.35)	3	Dust exposure, TB history, radiological severity, smoking	Readmission risk increased with smoking, male sex, older age, COPD, hepatitis, and non-standard treatment
Chuang *et al.*, 2015, [33]	Retrospective case-control study	Taipei, Taiwan	n = 245; TB patients with culture-positive sputum (71.4% male); age range: (16, 94)	Self-reported status	1.36 (1.03–2.36)	2	Age, BCG, smoking, site	Smoking negatively affected TB outcomes and treatment duration; cessation should be promoted during TB care
Liu *et al.*, 2023, [34]	Retrospective cohort	Hangzhou, Zhejiang Province, China	n = 1213; hospitalised patients confirmed positive for MTB (62.1% male); median age: 50–55	≥20 packs or ≥1/day for ≥1 year	1.95 (1.12–3.41)	4	Radiograph abnormality, age, sex, smoking, alcohol	Smoking increased discordance between QFT and MTB diagnosis, especially in elderly patients
Jiang *et al.*, 2024, [35]	Prospective cohort	Zhenjiang, Jiangsu Province, China	n = 39 122; elderly aged ≥65 years (46.1% male); age range: (65, +)	≥1 cigarette/day for ≥1 year; ex = quit ≥1 year	1.00 (0.60–1.66) *	7	Smoking status, age, sex	TB incidence remains high in the elderly; targeted screening is needed, especially in male, low-BMI, and ex-smoker groups
Tse *et al.*, 2018, [36]	Historical cohort study	Hong Kong SAR	n = 3185; Chinese male silicotic workers (100.0% male); mean age: 55	≥1/day for ≥6 months; ex = quit ≥3 months	NA	33	Smoking, lung cavitation, retreatment, sputum, age	Lung cancer mortality halved after smoking cessation; stronger benefits in small opacities and surface workers
Cao *et al.*, 2025, [37]	Multicenter longitudinal cohort	Zhongmu (Henan), Danyang (Jiangsu), China	n = 5924; rural residents ≥18 years (48.0% male); age range: (18, +)	Current, former, never (modelled separately)	NA	10	Education, alcohol, smoking initiation age	Smoking cessation and targeted screening can support TB control
Zhu *et al.*, 2024, [38]	Prospective cohort study	Zhejiang Province, China (Changshan and Jiangshan Counties)	n = 667; older adults (age ≥65 years) with LTBI (57.9% male); age range: (65, +)	Categories with pack-years noted	0.44 (0.07–2.80)*	2	SES, TB history, smoking, environmental exposure	Older adults with abnormal chest radiographs should be prioritized for TB preventive treatment. Smoking was not a significant risk factor in this LTBI cohort
Lin *et al.*, 2021, [19]	Prospective longitudinal study	Jiangxi Province, China	n = 634; successfully treated TB patients (69.9% male); mean age: SD = 44.5–16.9 years	Non = <20 packs or <1/day; ex = quit ≥7 days; current = ≥20 packs or 1/day	2.18 (1.65–2.88)*	7	Smoking, passive smoking, and industrial exposure	Patients between 34–73 and current smokers were at higher risk of recurrence; post-treatment monitoring and smoking cessation should be integrated in TB programmes
Leung *et al.*, 2015, [39]	Prospective cohort study	Hong Kong SAR	n = 16 345; TB patients under DOTS, excluding drug-resistant and non-residents (91.3% male); mean age: 47 (never smoker); 64 (ex-smoker); 50 (current smoker)	≥20 packs lifetime or ≥1/day for ≥1 year	1.63 (0.45–5.93)*	11	TB symptoms, alcohol, income, fuel use	Smoking worsened TB prognosis; cessation should be a core part of TB programmes
Wang *et al.*, 2009, [40]	Case-control and follow-up observational study	Yangzhong and Wujin County, Jiangsu Province, China	n = 613; adults from rural communities (74.7% male); mean age: SD = 56.0–16.5 year	Ever smoked ≥1 cigarette/day for ≥1 year; current = smoking or quit <1year; ex = quit ≥1year	1.93 (1.51–2.48)	1.35	Smoking, comorbidities, TB outcome	Smoking significantly increased TB risk, relapse risk, and reduced treatment adherence
Lin *et al.*, 2008, [41]	Modelling study	China, Jiangsu, Guizhou, Shanghai	General adult Chinese population	Smoked ≥1 cigarette/day for ≥6 months; ex-smoker = quit ≥3 months	NA	4	Age, smoking, TB symptoms	Eliminating smoking and solid fuels can reduce TB incidence by up to 71%, depending on DOTS; 26 million COPD and 6.3 million lung cancer deaths preventable
Chan-Yeung *et al.*, 2006, [42]	Cross-sectional retrospective study	Hong Kong SAR	n = 3682; residents in old age homes (27.6% male); age range: (51, +)	Current, former, and never smokers modelled separately	2.58 (1.79–3.71)	30	SES, smoking, HIV, fuel use	The study found that high latent TB prevalence was presented in elderly homes, and early detection and smoking cessation was needed

aOR – adjusted odds ratio, BCG – Bacillus Calmette-Guerin, BMI – body mass index, CCI – Charlson comorbidity index, COPD – chronic obstructive pulmonary disease, HBV – hepatitis B virus, DOTS – directly observed treatment, short-course, HCG – human chorionic gonadotropin, HIV – human immunodeficiency virus, LTBI – latent tuberculosis infection, MTB – Mycobacterium tuberculosis, NA – not available, PTB – pulmonary tuberculosis, QF+ -- QuantiFERON-TB Gold positive, SAR – Special Administrative Region, SES – socioeconomic status, TB – tuberculosis, VDD – vitamin D deficiency

*Studies with information on HRs.

The average age of participants ranged from the mid-30s to late 50s, and smoking exposure was typically defined as current or former use, with several studies quantifying pack-years. On average, the reported effect estimates indicated a 1.5-2.5-fold increased risk of TB among smokers, consistent with the pooled results (**Table 1**).

The proportion of male participants ranged from 0.0% [17] to 100.0% [36], with a mean of 63.5%. There were several studies focused specifically on older populations, including those aged ≥65 years (**Table 1**) [17,28,43]. The definition of smoking exposure varied, but most commonly included ≥1 cigarette/d for ≥1 year or ≥20 packs lifetime, and traditional cigarette consumption only. Some studies further analysed current, former, and never smokers, while a few included additional measures such as smoking intensity, pack-years, or cessation history. As for the follow-up durations, they ranged from 0.25 years [29], to 33 years [36], with a median of 3 years (interquartile range = 1–7). Moreover, the adjustment factors varied but frequently included age, sex, socioeconomic status, comorbidities, alcohol use, and passive smoking. Several studies also controlled for HIV status, radiographic severity, and occupational exposures. While most studies (n/N = 22/28; 78.6%) reported a positive association between smoking and TB risk or adverse outcomes, three (10.7%) found null associations [24,31,36], and 1 study (3.6%) suggested a possible protective effect [38]. Most of the included studies were prospective and observational cohort studies, accounting for approximately 42% of all included studies (**Figure 2**, Panel B).

### Results of meta-analysis

The reported ORs linking smoking and TB outcomes ranged widely from 0.28 [18] to 5.40 [19], suggesting a substantial variability in the reported associations. Most of the studies (10 out of 17) showed a statistically significant increased risk of TB among smokers (OR > 1 with 95% CI not crossing 1). Three studies [18,20,25] suggested a reduced or neutral risk (OR ≤ 1), though only Wei *et al.* and Leung *et al.* show statistically significant reductions [18,20]. Lin *et al.* (OR = 5.40; 95% CI = 3.02–9.66) and Ko *et al.* (OR = 4.70, 95% CI = 1.50–14.71) reported the highest risks, indicating that smoking behaviour may quintuple TB risk in certain populations [17,24]. While Wei *et al.* (OR = 0 · 28; 95% CI = 0.12–0.66) and Leung *et al.* (OR = 0.72; 95% CI = 0.64–0.81) are outliers, potentially influenced by confounding factors such as vaccination rates and access to healthcare.

Furthermore, Lin *et al.* [19] reported the highest risk (HR = 10.42; 95% CI = 2.97–36.63), indicating smokers had over 10 times the hazard of TB compared to non-smokers. Lin *et al.* [24] and Leung *et al.* [39] also showed significant increases in TB risk (HR = 5.40; CI = 3.02–9.67 and HR = 2.87; 95% CI = 2.00–4.11, respectively). While Jiang *et al.* [43] and Zhu *et al.* [38] found no statistically significant association (HR = 1.00; 95% CI = 0.60–1.66 and HR = 0.44; 95% CI = 0.07–2.80, respectively). Meanwhile, the pooled random-effects estimate (HR = 2.39; 95% CI = 1.28–4.45) indicated that smokers have about 2.4 times higher hazard of TB.

**Figure 3:**
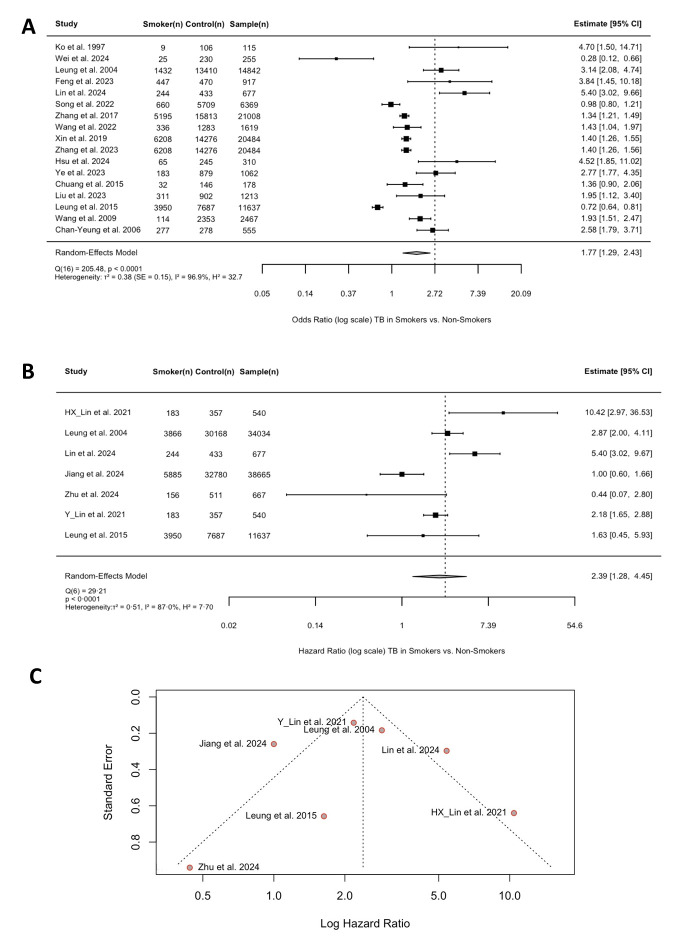
Random-effects meta-analysis of odds ratios and hazard ratios, and publication bias assessment for smoking behaviour and tuberculosis incidence. **Panel A.** Forest plot comparing the ORs of TB incidence between smokers and non-smokers across 17 studies. The forest plot shows the information on the ORs for the association between smoking behaviour and TB incidence from a random-effects meta-analysis of 17 included studies. The reference group is non-smokers. Each study is represented by a square and a horizontal line (95% CI). The size of the square corresponds to the study’s weight in the meta-analysis. The pooled summary estimate is represented by a diamond. **Panel B.** Forest plot of hazard ratios for tuberculosis incidence in smokers *vs.* non-smokers from seven included studies. The forest plot shows the information on the HRs for the association between smoking behaviour and TB incidence from a random-effects meta-analysis of seven included studies. The reference group is non-smokers. Each study is represented by a square and a horizontal line (95% CI). The size of the square corresponds to the study’s weight in the meta-analysis. The pooled summary estimate is represented by a diamond. **Panel C.** Funnel plot of the included articles with information on HR–publication bias. Funnel plot of log HRs against standard error for the studies included in the meta-analysis of the smoking behaviour and TB incidence. The vertical solid line represents the pooled effect estimate. The dashed lines represent the expected 95% CI limits for a given standard error, assuming no heterogeneity or bias.

Both forest plots supported a positive association between smoking behaviour and TB, with most studies in each showing elevated risk (**Figure 3**, Panels A and B). The pooled estimates of the two forest plots align closely with each other, as the pooled OR was 1.77 with 95% CI = 1.29–2.43, and the pooled HR was 2.39 with 95% CI = 1.28–4.45.

The pooled estimate indicated an overall elevated risk of TB among smokers. However, the forest plot revealed considerable heterogeneity across studies, with ORs ranging from 0.28 to 5.40 and HRs ranging from 0.44 to 10.42, both showing partially overlapping 95% CIs (**Figures 3**, Panels A and B). This variability may be attributed to differences in study design, population characteristics such as smoking intensity, TB diagnostic criteria, and geographic locations.

### Quality of reporting

The overall quality of the seven studies reporting HR data was mixed, as reflected by the asymmetric distribution and the gap observed in the bottom-right quadrant (**Figure 3**, Panel C). Most studies were of high quality, with well-selected cohorts, though some limitations were present, particularly due to confounding factors such as variability in study settings (Figures S1 and S2 in the **Online Supplementary Document**).

## DISCUSSION

This systematic review and meta-analysis of 29 studies conducted across China provides robust evidence that smoking substantially increases the risk of developing active TB. Our pooled estimates show that smokers had 77% higher odds (OR = 1.77; 95% CI = 1.29–2.43) and a 2.4-fold higher hazard (HR = 2.39; 95% CI = 1.28–4.45) of TB incidence compared with non-smokers. Importantly, more than half of the included studies reported statistically significant positive associations, and the consistency between ORs- and HRs-based syntheses strengthens confidence in this relationship. We observed significant variability across individual studies, with some reporting exceptionally high risks (up to 10-fold), while others, such as Wei *et al.* [18] and Leung *et al.* [20], suggested neutral or even protective effects. These outliers are likely due to contextual factors, such as differences in vaccination coverage, healthcare access, or unmeasured confounding variables in those studies. Geographically, most studies were conducted in the eastern region of China, likely reflecting regional disparities in research capacity and awareness of smoking-related health risks, with fewer studies emerging from the western region’s provinces, where the TB burden is higher [18–20]. Available data indicate that provinces in southern China have relatively higher male smoking prevalence compared with other regions. Yunnan, Guizhou, and Hunan, which ranked highest in *per capita* cigarette production in 2018, also reported the highest smoking rates. However, the variation in smoking prevalence across provinces is less pronounced than the sharp upward trend observed among adult men [25]. Overall, the findings reinforce smoking as a significant and modifiable risk factor for TB in China, that built on previous study, while also emphasising heterogeneity that future research must address more closely.

Previous global reviews have consistently reported smoking as a significant risk factor for TB incidence [44], but most have aggregated data from heterogeneous settings and have limited their applicability to high-burden countries such as China [40,45]. In our study, we addressed a crucial gap in the literature by focusing exclusively on Chinese populations, particularly studies conducted after the COVID-19 period, to generate population-specific estimates that are highly relevant to public health programming in China and on a broader global scale [21,46]. Although evidence remains mixed, the COVID-19 pandemic may have indirectly exacerbated smoking-related risks through disruptions to TB services, heightened psychosocial stress, and shifts in smoking behaviours, with potential implications for TB risk and outcomes.

Our analysis aligns with global evidence demonstrating the connection between tobacco use and increased TB risk, while also emphasising variations in effect size and specific population subgroups that are particularly relevant in China. This is especially significant given that China has the world's largest population of smokers and one of the highest TB burdens; the convergence of the burdens represents a dual public health crisis. Our findings underscore the importance of integrating tobacco control efforts with TB prevention, treatment services, and other disease management strategies to enhance TB control. Evidence-based cessation strategies, ranging from community-level interventions to policy-level measures such as taxation and smoke-free legislation, could therefore have a significant impact on TB incidence reduction. For example, task sharing has been found effective in managing diseases to alleviate and assign the workload of physicians and pharmacists [47,48].

This body of evidence also highlights several key points of controversy that should be considered when interpreting the findings of this study. Some included studies suggest a bidirectional relationship, where TB patients are more likely to start or continue smoking, complicating efforts to establish clear causal links. Our review emphasises the importance of disentangling temporality and underlying mechanisms through prospective cohort studies. Variations in effect sizes compared to previous global reviews may stem from contextual and methodological differences, reinforcing the importance of region-specific evidence [49]. Just as tailored strategies were required to enhance public health engagement [13,50], regionally focused interventions are crucial for effective tobacco and TB control efforts in China.

In addition to active smoking, passive smoking exposure in household and workplace settings may also contribute to TB risk and potentially modify observed associations between active smoking and TB incidence. Several included studies did not systematically assess second-hand smoke exposure, raising the possibility of residual confounding, particularly in high-prevalence settings where non-smokers may experience substantial involuntary exposure. As a result, the estimated effects attributed to active smoking may partially reflect combined exposure pathways within shared domestic or occupational environments. However, the available data do not allow for formal stratified or interaction analyses by passive smoking exposure, and future studies should explicitly measure and model both active and passive tobacco exposure. Future research should also focus on prospective, well-designed cohort studies that use standardised definitions for both smoking exposure and TB outcomes, as well as monitor trends in smoking behaviours. The impact of innovative products like e-cigarettes on smoking habits should also be carefully investigated [51]. Dose-response analyses are essential to better understand the relationship between smoking intensity and TB risk, as well as the potential benefits of smoking cessation at various stages of disease progression [52]. Additionally, intervention studies are needed to assess the effectiveness of integrating smoking cessation support into TB care pathways, in both clinical and community settings, to improve disease management. Finally, it is crucial to address equity-related factors, such as urban-rural disparities and socioeconomic inequalities, that could exacerbate the dual burden of smoking and TB in China [53].

Based on the available evidence, we indicate that smoking is not merely an incidental association but a significant and modifiable risk factor for TB incidence in Chinese populations. From a clinical perspective, this underscores the importance of routinely assessing smoking history and providing cessation counselling as part of TB care [54]. At the policy level, our findings highlight the opportunity for synergistic gains by aligning tobacco control initiatives with China’s End TB strategy. Evidence from other settings with comparable TB and tobacco burdens shows that integrating risk factor management into routine health services can be both feasible and effective and reinforces the potential for tobacco control to act as a lever for TB reduction in China [55–58]. In regions with high tobacco use prevalence and substantial TB burden, coordinated action on these interconnected risk pathways is crucial for accelerating progress toward national and global TB elimination goals.

### Limitations

Our analysis focused exclusively on Chinese populations and thus addressed gaps in prior meta-analyses that aggregated data across heterogeneous settings. We performed sensitivity analyses and risk-of-bias assessments to evaluate the robustness of the findings regarding socioeconomic factors and occupational exposures, which are independently associated with both smoking and TB [59]. Exposure definitions varied across studies, ranging from ever/never smokers to pack-years or smoking intensity, which may have contributed to heterogeneity. Similarly, variation in TB diagnostic criteria across studies may have contributed to between-study heterogeneity, although most studies relied on standardised clinical or programmatic definitions in routine practice. Moreover, the inconsistent reporting of sex- and age-stratified results limited our ability to thoroughly investigate differences across subgroups. Lastly, the possibility of publication bias cannot be ruled out, particularly considering the common tendency to underreport null findings.

## CONCLUSIONS

Smoking remains a major and preventable contributor to TB incidence in China. We found that smokers face substantially higher risks of developing active TB, with consistent evidence across study designs and effect measures. The findings underscore smoking as a critical, yet under-addressed, determinant of TB burden in a setting with both high tobacco use and persistent TB transmission. Integrating tobacco control into TB prevention and care offers a practical opportunity to accelerate progress toward national and regional TB elimination targets. Aligning smoking cessation with TB programmes could yield meaningful gains in disease reduction, particularly in high-risk populations. Addressing these converging epidemics is essential for strengthening TB control efforts in China and across the Western Pacific region.

## Additional material


Online Supplementary Document
